# RNA N6-methyladenosine demethylase FTO promotes pancreatic cancer progression by inducing the autocrine activity of PDGFC in an m^6^A-YTHDF2-dependent manner

**DOI:** 10.1038/s41388-022-02306-w

**Published:** 2022-04-14

**Authors:** Zhen Tan, Si Shi, Jin Xu, Xiaomeng Liu, Yubin Lei, Bo Zhang, Jie Hua, Qingcai Meng, Wei Wang, Xianjun Yu, Chen Liang

**Affiliations:** 1 Department of Pancreatic Surgery, Fudan University Shanghai Cancer Center, Shanghai, 200032 China; 2 Department of Oncology, Shanghai Medical College, Fudan University, Shanghai, 200032 China; 3 Shanghai Pancreatic Cancer Institute, Shanghai, 200032 China; 4 Pancreatic Cancer Institute, Fudan University, Shanghai, 200032 China

**Keywords:** Transcriptomics, Oncogenes

## Abstract

RNA N6-methyladenosine (m^6^A) is an emerging regulator of mRNA modifications and represents a novel player in tumorigenesis. Although it has functional significance in both pathological and physiological processes, the role of m^6^A modification in pancreatic ductal cancer (PDAC) remains elusive. Here, we showed that high fat mass and obesity-associated gene (FTO) expression was associated with a poor prognosis in PDAC patients and that suppression of FTO expression inhibited cell proliferation. Here, m^6^A sequencing (m^6^A-seq) was performed to screen genes targeted by FTO. The effects of FTO stimulation on the biological characteristics of pancreatic cancer cells, including proliferation and colony formation, were investigated in vitro and in vivo. The results indicate that FTO directly targets platelet-derived growth factor C (PDGFC) and stabilizes its mRNA expression in an m^6^A-YTHDF2-dependent manner. m^6^A-methylated RNA immunoprecipitation-qPCR (MeRIP-qPCR), RNA immunoprecipitation (RIP), and luciferase reporter assays were employed to validate the specific binding of FTO to PDGFC. PDGFC upregulation led to reactivation of the Akt signaling pathway, promoting cell growth. Overall, our study reveals that FTO downregulation leads to increased m^6^A modifications in the 3ʹ UTR of PDGFC and then modulates the degradation of its transcriptional level in an m^6^A-YTHDF2-dependent manner, highlighting a potential therapeutic target for PDAC treatment and prognostic prediction.

## Introduction

Pancreatic ductal adenocarcinoma (PDAC) is a solid malignancy with the poorest prognosis and was the seventh leading cause of cancer death worldwide in 2018. The 5-year patient survival rate at the time of diagnosis is approximately 10%. Although a small subset of patients is diagnosed with a localized or resectable tumor, the 5-year survival rate is only 20% after surgery [1]. Accumulating evidence suggests that PDAC is resistant to the most effective systemic agents and antineoplastic therapies and has a low response rate and a rapid progression rate [2]. Therefore, it is urgent to explore the mechanisms underlying PDAC progression and to develop novel treatments for this disease.

It is widely known that genetic and epigenetic regulation can modulate cell growth and division [3, 4]. RNA epitranscriptomics has recently received increased attention and interest in the research community. Of greater than 100 known RNA modifications, N6-methyladenosine (m^6^A), namely, methylation of the adenosine base at the nitrogen-6 position of mRNA, is the most pervasive internal mRNA modification [5–7]. Gene examination technology has demonstrated that m^6^A modification is enriched in 3ʹ untranslated regions (UTRs), is translated near the 5ʹ UTR in long exons and has a consensus sequence represented by RRACH (R = G or A; H = A, C, or U) [8]. In most eukaryotic species and viral mRNAs, m^6^A with conserved sequences plays an essential role at the mRNA processing, transcriptional and posttranscriptional levels and in metabolism. m^6^A modifications, such as DNA and histone methylation, are reversible and dynamic processes mediated by “writers” (RNA methyltransferases), “erasers” (demethylases) and “readers” (m^6^A-binding proteins) [9, 10]. Demethylation, which involves the disposal of methyl groups, is employed by the demethylase enzyme family, which principally includes AlkB homolog H5 (ALKBH5) and fat mass and obesity-associated protein (FTO). FTO, which belongs to these alpha-ketoglutarate-dependent dioxygenase families, catalyzes m^6^A into Fe (II) and α-ketoglutaric acid-dependent forms. FTO became notable in genome-wide association studies because single nucleotide polymorphisms located in its genomic locus are associated with obesity [11, 12]. Furthermore, large-scale epidemiological studies have shown that FTO is closely associated with the development of cancers, such as breast and prostate cancers, leukemia, lymphoma and myeloma [13–15]. Previous study observed that FTO knockdown could suppressed pancreatic cell proliferation and DNA synthesis in PDAC [16]. However, the definitive role of FTO in concrete mechanisms behind m^6^A-dependent regulation on PDAC remains elusive.

In the present study, we aimed to determine the role of m^6^A modification in PDAC and investigated the underlying actuation mechanism toward modification that affects PDAC progression. We first demonstrated the function of FTO in facilitating PDAC progression and correlated it with survival outcomes in PDAC patients. Next, we found that platelet-derived growth factor C (PDGFC) is the target gene of FTO and is regulated in an m^6^A-YTH domain family 2 (YTHDF2)-dependent manner. Hence, we demonstrate that FTO is an oncogene and could serve as a therapeutic target for PDAC patients.

## Results

### FTO is highly expressed in PDAC and associated with a poor prognosis

**Fig. 1:**
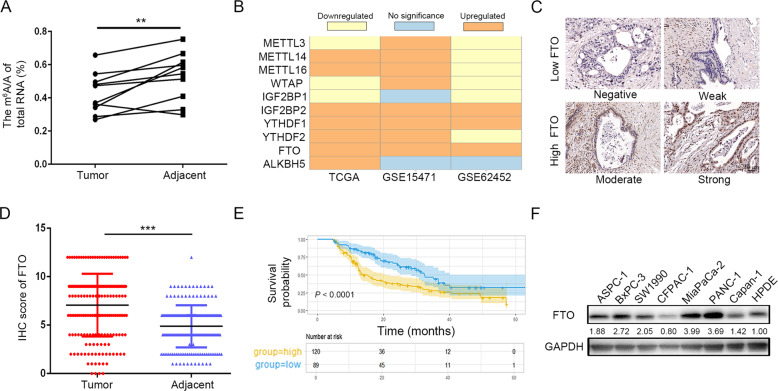
Increased FTO expression in human pancreatic cancer. **A** Global mRNA m^6^A levels in human pancreatic cancer samples determined by RNA m^6^A colorimetric analysis. (*P* < 0.05). **B** FTO was constantly overexpressed in PDAC in the TCGA-GTEx cohort and two GEO datasets. **C** Representative images of IHC staining for FTO in TMAs (scale bar, 50 µm). **D** FTO expression in PDAC and adjacent normal tissues, as determined by the IHC score (*P* < 0.001). **E** The OS of PDAC patients was assessed using Kaplan–Meier analysis based on FTO expression (*n* = 209, *P* < 0.0001). **F** Western blot analysis of FTO expression in PDAC cells and HPDE cells.

To interpret the key roles of m^6^A in PDAC, we examined global m^6^A levels in 10 fresh human pancreatic tumor tissues and their corresponding normal tissues via m^6^A colorimetric analysis. Upon initial observation, a notable decrease in global m^6^A abundance was observed in PDAC tissues compared with corresponding normal tissues (Fig. 1A). Then, to evaluate the expression profiles of the major m^6^A-modifying enzymes in PDAC, we analyzed clinical datasets from The Cancer Genome Atlas (TCGA), GTEx, GSE15471, and GSE62452 and found that FTO mRNA expression levels were significantly elevated in PDAC tissues compared to those in normal tissues (Fig. 1B). Furthermore, we validated the bioinformatics data in the Fudan University Shanghai Cancer Center (FUSCC) cohort. Immunohistochemistry (IHC) staining was performed on tissue microarrays (TMAs) containing samples from 209 patients (Fig. 1C). Based on the IHC score, FTO was expressed at higher levels in tumor tissues compared with normal tissues (Fig. 1D). Kaplan–Meier survival curves revealed that high FTO expression was associated with a poor prognosis of PDAC patients (*P* < 0.0001; Fig. 1E). Moreover, univariate and multivariate Cox regression analyses showed that FTO was an independent prognostic factor in PDAC, where higher FTO expression was predictive of a short overall survival (OS) rate for PDAC patients (Table 1). Finally, we found that FTO protein expression was upregulated in multiple PDAC cell lines compared with human pancreatic duct epithelial (HPDE) cells (Fig. 1F).

**Table 1:** Univariate and multivariate Cox regression of overall survival for patients with PDAC.

	Univariate			Multivariate		
Characteristics	HR	95% CI	*P*	HR	95% CI	*P*
Age,y						
<60	1	0.83092–1.7503	0.32443			
≥60	1.206					
Gender						
Female	1	0.49109–1.018	0.062298			
Male	0.70705					
Tumor differentiation						
Well/Moderate	1	1.3255–2.8431	0.006257	1	1.0663–2.341	0.0226
Poor	1. 6853			1.58		
Stage						
I–IIa	1	1.1592–2.45	0.028616	1	1.0791–2.384	0.01947
IIb–IV	1. 5249			1.604		
FTO expression						
Low	1	1.0451–2.225	0.000656	1	1.2659–2.775	0.0017
High	1. 9413			1.8742		

*CI* Indicates confidence interval, *HR* Hazard ratio.

Univariate *P* values were derived with log-rank test. Multivariate *P* values were derived with Cox regression analysis.

### Silencing FTO inhibited PDAC tumor growth

**Fig. 2:**
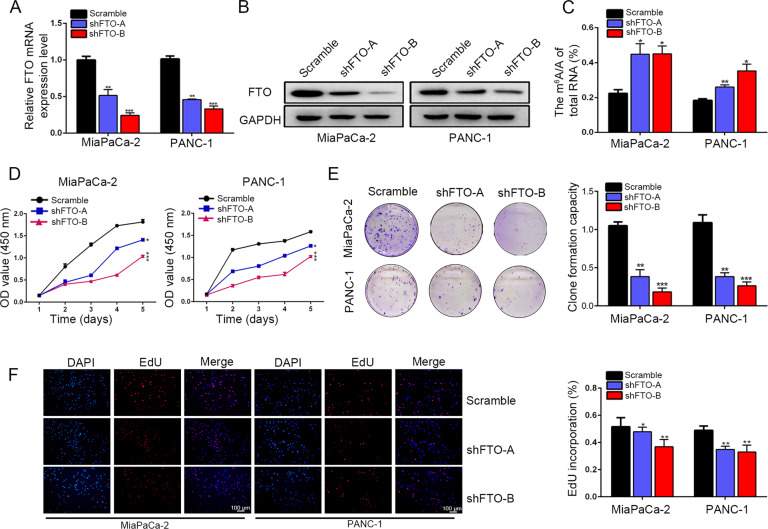
FTO significantly promoted pancreatic cancer progression. **A**, **B** Knockdown of FTO was verified at both the mRNA and protein levels. **C** Global mRNA m^6^A levels in FTO knockdown cells were detected by RNA m^6^A colorimetric analysis. **D** Silencing FTO expression decreased proliferation, as reflected by the CCK-8 proliferation assay results. **E** Decreased FTO expression inhibited the colony formation capacity of PANC-1 and MiaPaCa-2 cells. **F** EdU incorporation assays showed the effects of FTO knockdown on cell proliferation.

To investigate the roles of FTO in PDAC, PANC-1 and MiaPaCa-2 cells were transfected with two stable FTO knockdown constructs with a lentivirus encoding short hairpin RNAs (shFTO-A and shFTO-B). The FTO knockdown effects were verified at both the mRNA and protein expression levels (Fig. 2A, B). Then, CFPAC-1 cells with the lowest level of FTO expression was transfected with an FTO-encoding lentivirus and empty vectors respectively (Supplementary Fig. S1A). Interestingly, FTO knockdown resulted in increased m^6^A levels (Fig. 2C). FTO knockdown significantly decreased PDAC cell proliferation (Fig. 2D) and decreased the colony formation efficiency compared to that of the scramble groups (Fig. 2E). Similarly, we verified the inhibitory effect of FTO knockdown on cell proliferation using a 5-ethynyl-2’-deoxyuridine (EdU) staining assay. EdU staining assays revealed that FTO knockdown greatly decreased the percentages of EdU-positive cells (Fig. 2F). In contrast, FTO overexpression led to an increased cell proliferation rate in the CFPAC-1/oeFTO group compared to that of the control groups (Supplementary Fig. S1B–D).

**Fig. 3:**
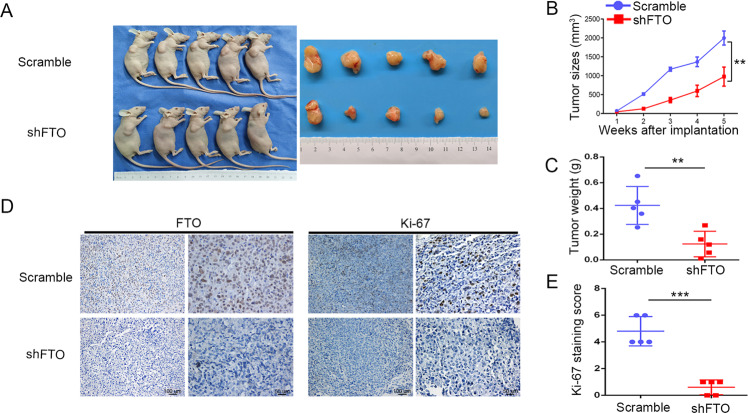
Silencing FTO inhibited pancreatic tumor growth in nude mouse models. **A** MiaPaCa-2 cells stably transfected with FTO shRNA (shFTO-B) or scramble shRNA were subcutaneously inoculated into nude mice. **B** Tumor growth curves were constructed based on the tumor volumes tested using Vernier calipers every week. **C** The relative weights of tumors were measured on the 5th week after subcutaneous transplantation. **D**, **E** The expression of FTO and the proliferation marker Ki-67 was determined in tumor tissue sections from the xenografts using IHC.

Next, we implanted a subcutaneous pancreatic xenograft tumor to further determine the oncogenic roles of FTO in vivo. MiaPaCa-2 cells with stable FTO knockdown (shFTO-B) were subcutaneously injected into 4-week-old female BALB/c nude mice (Fig. 3A). Consistent with in vitro observations, tumor growth size and weight were significantly decreased in FTO silenced groups compared with scrambled groups (Fig. 3B, C). As shown in Fig. 3D, E, subsequent IHC staining using antibodies against FTO and the proliferation marker Ki-67 showed that FTO knockdown significantly reduced Ki-67 staining in the shFTO group compared with the scrambled group. Hence, FTO plays a critical role in promoting PDAC tumor growth.

### Identification of FTO downstream targets by m^6^A-Seq

**Fig. 4:**
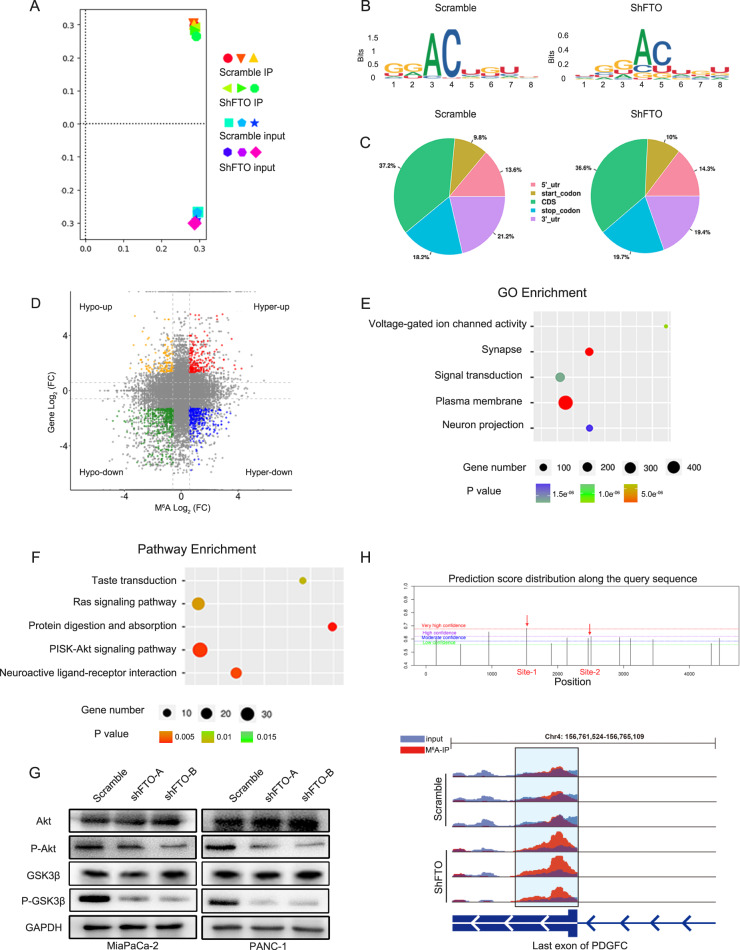
m^6^A methylation underlies the effects of FTO. **A** Principal component analysis on m^6^A-Seq data. **B** Top consensus motif analysis of m^6^A-Seq peaks in scramble and shFTO MiaPaCa-2 cells. **C** Proportion of m^6^A peak distribution in the 5ʹ UTR, start codon, CDS, stop codon or 3ʹ UTR of mRNA transcripts. **D** Distribution of peaks (Fold change > 1.5 or < −1.5, *P* < 0.05) with a significant change in the mRNA expression level and m^6^A level. **E** A cluster profiler identified the enriched gene ontology processes of DEGs. **F** Enrichment of an Akt signaling gene expression signature by KEGG analysis. **G** Western blotting to measure Akt, P-Akt, and P-GSK3β expression levels in transformed PANC-1 and MiaPaCa-2 cells described above. **H** IGV analysis showed that FTO attenuation increased m^6^A modification levels of PDGFC mRNA.

To identify FTO downstream targets in PDAC, we performed methylated RNA immunoprecipitation sequencing (MeRIP-seq) to map the m^6^A modification. Principal component analysis (PCA) demonstrated that three repeats of each sample clustered together, indicating good reproducibility among the two groups (Fig. 4A). The GGAC motif was highly enriched within m^6^A sites in both scramble and FTO knockdown cells (Fig. 4B). According to the m^6^A-seq results, we further found that m^6^A peaks were enriched mainly in the adjacent coding sequence (CDS) and 3ʹ UTR (Fig. 4C). We further investigated MeRIP-seq expression data and explored the proportion of peaks with a significant change at both the m^6^A and RNA levels. Indeed, 292 genes with hypermethylated m^6^A peaks along with decreased mRNA expression were detected in the two groups (Fig. 4D). Among these differentially expressed genes (DEGs), voltage-gated ion channel activity, signal transduction and plasma membrane were the most enriched terms in the Gene Ontology (GO) enrichment analysis (Fig. 4E). Kyoto Encyclopedia of Genes and Genomes (KEGG) enrichment analyses revealed that the PI3K/Akt pathway-enriched signature was related to FTO-dependent transcription in PDAC (Fig. 4F). To validate this finding, we first examined the expression of Akt, phosphorylated Akt (P-Akt) and phosphorylated GSK3β (P-GSK3β) in cells transfected with the scramble control and in FTO knockdown cells and xenografts (Fig. 4G and Supplementary Fig. S2A, B). FTO knockdown markedly decreased the phosphorylation levels of these genes in PANC-1 and MiaPaCa-2 cells. We selected and validated meRIP-seq identified differentially expressed genes (DEGs) enriched in the PI3K/Akt pathway. Besides it, a collective of genes related with m^6^A modification and previously reported influence PI3K-Akt signaling was also validated [17, 18] (Supplementary Fig. S3). However, the PDGFC expression difference between the scramble and FTO knockdown groups in MiaPaCa-2 and PANC-1 cells is the largest. Most importantly and intriguingly, in the FTO knockdown groups, some downregulated transcripts exhibited hypermethylated m^6^A modification, including PDGFC. We focused on PDGFC m^6^A peaks and detected enrichment around the 3ʹ UTR of its mRNA, which was increased upon FTO knockdown, as determined by integrative genomics viewer analysis (Fig. 4H). Hence, PDGFC was chosen as a candidate target of the FTO mediated m^6^A modification.

### FTO loss downregulated PDGFC mRNA levels in an m^6^A-YTHDF2-dependent manner

**Fig. 5:**
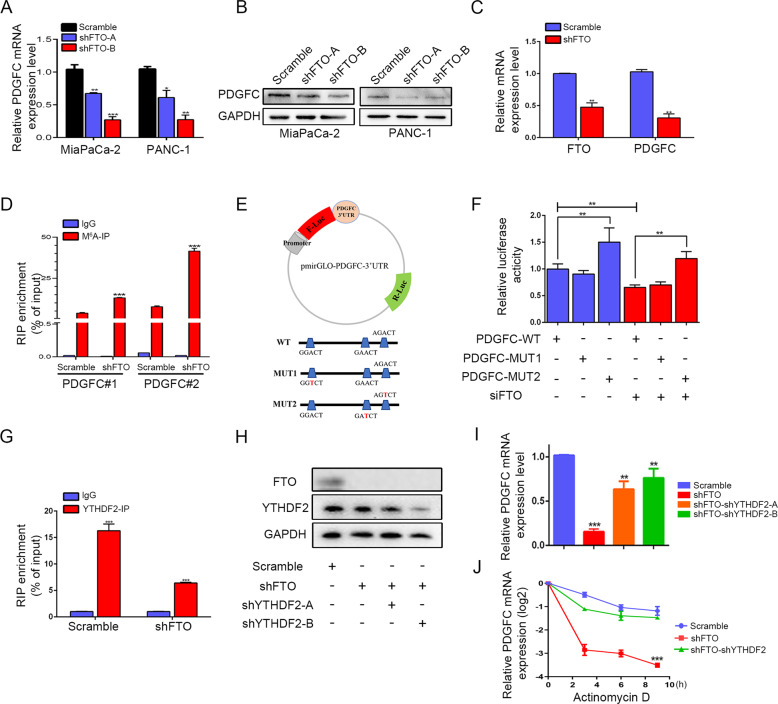
FTO regulates PDGFC mRNA levels in an m^6^A-YTHDF2-dependent manner. **A**, **B** FTO knockdown decreased PDGFC mRNA and protein levels. **C** PDGFC expression levels were determined in xenograft tumor tissue sections using qRT-PCR and IHC. **D** MeRIP-qPCR analysis confirmed that the m^6^A modification of PDGFC mRNA was enriched upon FTO knockdown. **E** Wild-type or m^6^A consensus sequence mutant PDGFC 3ʹ UTR was fused with firefly luciferase reporter. Mutation of m^6^A consensus sequences was generated by replacing adenosine with thymine. **F** Relative luciferase activity of the wild-type and mutant PDGFC 3ʹ UTR reporter vectors. **G** YTHDF2 was immunoprecipitated and then subject to qRT-PCR to assess PDGFC transcript levels. **H** Immunoblotting assay of YTHDF2 protein levels in MiaPaCa-2 cells with scramble, FTO knockdown and YTHDF2 knockdown. **I** qPCR analysis of PDGFC mRNA levels (scramble and YTHDF2 knockdown) in the absence or presence of FTO knockdown. **J** qPCR analysis of PDGFC mRNA levels (scramble and YTHDF2 knockdown) in the absence or presence of FTO knockdown after actinomycin D treatment.

FTO knockdown in MiaPaCa-2 and PANC-1 cells reduced PDGFC mRNA and protein levels (Fig. 5A, B). Not surprisingly, a similar trend was also validated in xenografts by quantitative real-time polymerase chain reaction (qRT-PCR) and IHC (Fig. 5C and Supplementary Fig. S2C). Consequently, to clarify whether FTO can directly regulate m^6^A methylation of PDGFC, MeRIP-qPCR was then performed to confirm the FTO-mediated m^6^A demethylation of PDGFC mRNA. Two potential m^6^A consensus sequence GGAC (RRACH) motifs, including PDGFC site-1 and site-2, were predicted by the m^6^A modification site predictor (https://www.cuilab.cn/sramp). As expected, FTO knockdown dramatically promoted m^6^A levels in PDGFC mRNA compared to the IgG-RIP group (Fig. 5D). To further explore the role of the m^6^A modification in PDGFC mRNA expression, we replaced N6-methylated adenosine (A) with thymine (T) at the two potential m^6^A sites of PDGFC mRNA and cloned them into the dual-luciferase reporter construct pmirGLO to generate reporter vectors. We transfected 293 T cells with small interfering RNA (siRNA) against FTO and control siRNA (Supplementary Fig. S2D). Then, 293 T cells were then cotransfected with dual luciferase reporter plasmids along with wild-type and 2 mutant PDGFC vectors. Compared with control cells, FTO knockdown cells exhibited significantly decreased luciferase activity. PDGFC-MUT2 almost abolished this induction, showing that the modulation of PDGFC expression was under the control of FTO associated m^6^A modification on MUT2 (Fig. 5F).

The m^6^A modification performs biological functions as a potent posttranscriptional modulator depending on the m^6^A “reader”, as it promotes mRNA translation efficiency or affects mRNA stability. As the first m^6^A reader to be discovered, YTHDF2 carries a conserved m^6^A-binding domain that can discern specific m^6^A sites, recruit the CCR4-NOT complex deadenylation mediator and deliver RNA to the processing body and eventually stimulate the degradation of m^6^A-modified RNA [19]. Hence, due to the specificity of YTHDF2 as the main m^6^A reader, we hypothesized that YTHDF2 functions at the level of RNA recognition and interaction in PDGFC. By evaluating TCGA and Gene Expression Omnibus (GEO) databases, we found that YTHDF2 was upregulated in PDAC tissues compared to normal tissues. IHC staining information on YTHDF2 in PDAC was obtained for our cohort consisting of 24 PDAC tissues also proved it (Supplementary Fig. S4). Indeed, YTHDF2 interacted strongly with PDGFC, as evaluated through RNA immunoprecipitation (RIP) assays. We found that YTHDF2 enrichment at PDGFC transcripts was significantly reduced when FTO was knocked down (Fig. 5G). Furthermore, we knocked down YTHDF2 in FTO reduced MiaPaCa-2 cells and observed partial recovery of PDGFC expression in pancreatic cells (Fig. 5H, I). Additionally, YTHDF2 knockdown also abrogated the stability of PDGFC mRNA decrease under FTO knockdown conditions (Fig. 5J). Thus, our data suggest that FTO modulates methylated PDGFC mRNA levels in an m^6^A-YTHDF2-dependent manner.

### PDGFC is a pro-oncogenic factor and is positively correlated with FTO expression in PDAC patients

**Fig. 6:**
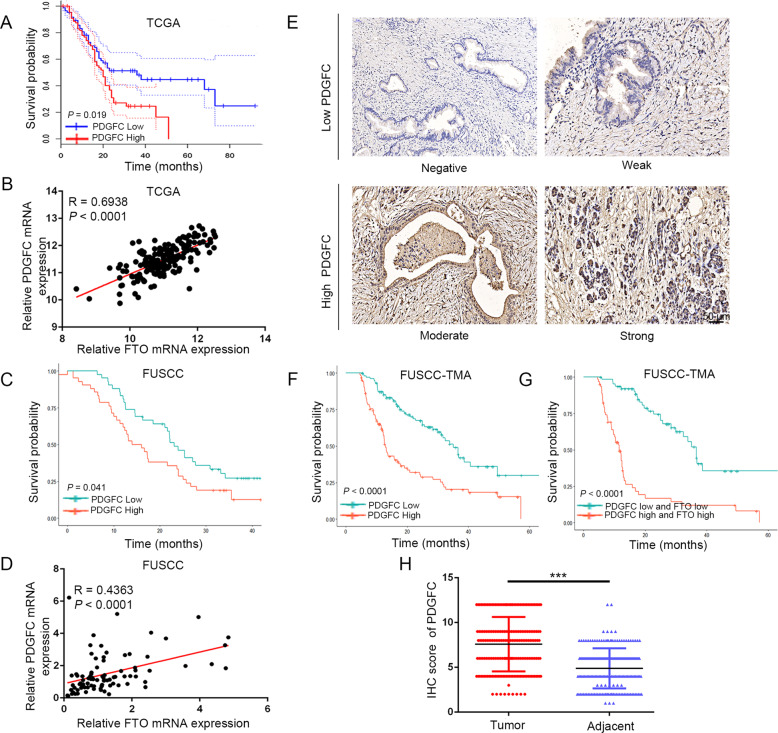
Clinical correlation between FTO and PDGFC in PDAC. **A** The expression data were divided into a high expression group and a low expression group based on the median. Kaplan–Meier analysis of the correlation between PDGFC levels and OS of pancreatic cancer patients in the TCGA cohort. **B** Pearson analysis of the correlation between the levels of PDGFC and FTO expression in TCGA cohort. **C** The expression data were divided into a high expression group and a low expression group based on the median. Kaplan–Meier analysis of the correlation between the PDGFC levels and OS of pancreatic cancer patients in the FUSCC cohort. **D** Pearson analysis of the correlation between PDGFC and FTO expression levels in the FUSCC cohort. **E** Representative images showing high or low expression of PDGFC in TMAs. **F** The OS of patients with PDAC was assessed using Kaplan–Meier analysis based on PDGFC expression. **G** PDGFC and FTO expression was stratified by the individual medians of IHC analysis, and the patients were divided into two groups as indicated. **H** PDGFC was significantly upregulated in PDAC tumor samples.

Evaluation of the TCGA and GEO databases revealed that PDGFC was upregulated in PDAC tissues compared to normal tissues. In addition, PDGFC expression was significantly increased in the inherited germline mutation groups compared with the wild-type group (Supplementary Fig. S5). Analysis of TCGA data revealed that PDGFC levels were negatively correlated with the OS of PDAC patients, and a significant positive correlation between PDGFC and FTO levels was observed (Fig. 6A, B). To validate their correlation, qRT-PCR was performed on pancreatic tumor tissues from FUSCC to measure PDGFC expression. Notably, our results showed that higher PDGFC levels were significantly correlated with worse OS (Fig. 6C) and positively correlated with FTO expression (Fig. 6D). Moreover, using IHC staining performed on TMAs, we further perform survival analyses of PDGFC and validated it expression differences between tumor and adjacent normal tissue (Fig. 6F, H). In addition, as shown in Fig. 6G, we also generated a new IHC panel containing PDGFC and FTO to predict the prognosis of PDAC. Kaplan–Meier survival analysis indicated that the patients with high expression of the two markers had the shortest OS.

### FTO-mediated activation of PDGFC is responsible for the tumorigenesis of PDAC

**Fig. 7:**
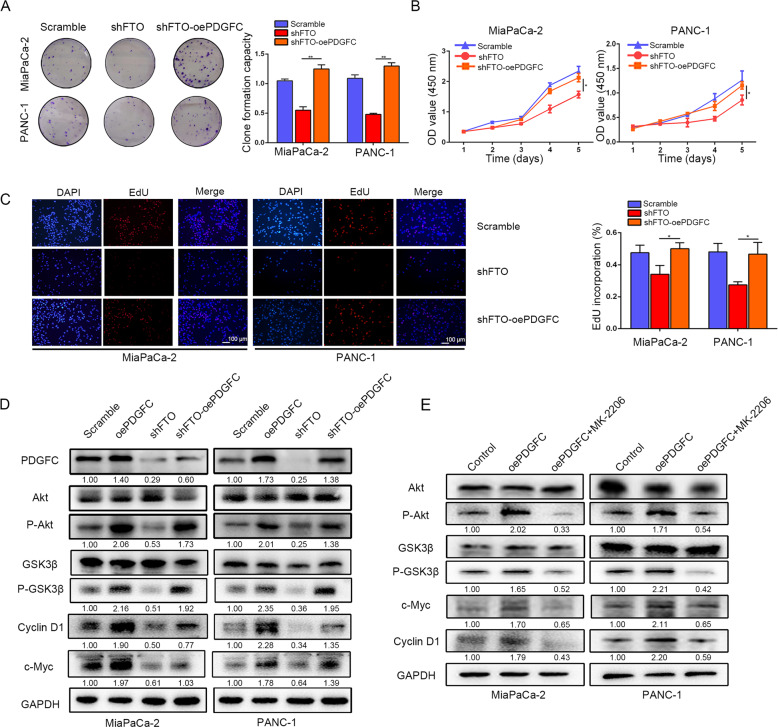
FTO maintains PDGFC expression in PDAC. **A** The colony formation capacity of PANC-1 and MiaPaCa-2 cells with FTO knockdown was partially rescued by PDGFC overexpression. **B** Overexpression of PDGFC rescued proliferative activity, as reflected by the CCK-8 proliferation assay. **C** EdU incorporation assays revealed that FTO significantly inhibits proliferative activity, which can be rescued by overexpression of PDGFC. **D** PDGFC protein levels were measured by western blot in MiaPaCa-2 and PANC-1 cells transfected with lentiviruses carrying shFTO and/or PDGFC; Akt downstream targets in MiaPaCa-2 and PANC-1 cells transfected with lentiviruses carrying shFTO and/or PDGFC were detected. **E** Levels of protein intermediates in the activated Akt pathway in PDGFC-overexpressing or control cells following simultaneous treatment with an Akt inhibitor (MK2206, 7 μM in MiaPaCa-2 cells and 12 μM in PANC-1 cells for 3 h) are shown.

To ascertain whether PDGFC mediates the function of FTO in PDAC growth and progression, we first transfected lentiviral vectors encoding human PDGFC inserts based on the endogenous expression of PDGFC into cells. Transfection efficiency and localization were determined by immunoblotting and immunofluorescence staining. Subsequently, cell counting kit-8 (CCK-8), colony formation, EdU were performed to determine the impact of PDGFC on the stimulation of tumorigenesis (Supplementary Fig. S6). We found that PDGFC overexpression particularly increased PDAC cell viability and colony formation but also abrogated the decrease under FTO knockdown conditions (Fig. 7A–C). Consequently, these results provide evidence that PDGFC overexpression is responsible for FTO-mediated tumor progression. Notably, the activated Akt pathway subsequently increased the expression of genes involved in proliferation and cell cycle pathways, including c-Myc and Cyclin D1. Akt/GSK3β phosphorylation and c-Myc and Cyclin D1 expression were assessed in pancreatic cancer cells. FTO knockdown decreased the expression of these genes, whereas PDGFC overexpression rescued this tendency (Fig. 7D). Moreover, to further confirm our hypothesis, we tested whether an Akt inhibitor (MK2206) could abolish the expression of these genes in PDGFC overexpressing cells. Akt/GSK3β phosphorylation levels and c-Myc and Cyclin D1 expression were upregulated in PDGFC overexpressing cells, whereas these levels were reduced in cells treated with MK2206 (Fig. 7E).

### FTO induces the autocrine activity of PDGFC and is associated with proliferation in PDAC cells

**Fig. 8:**
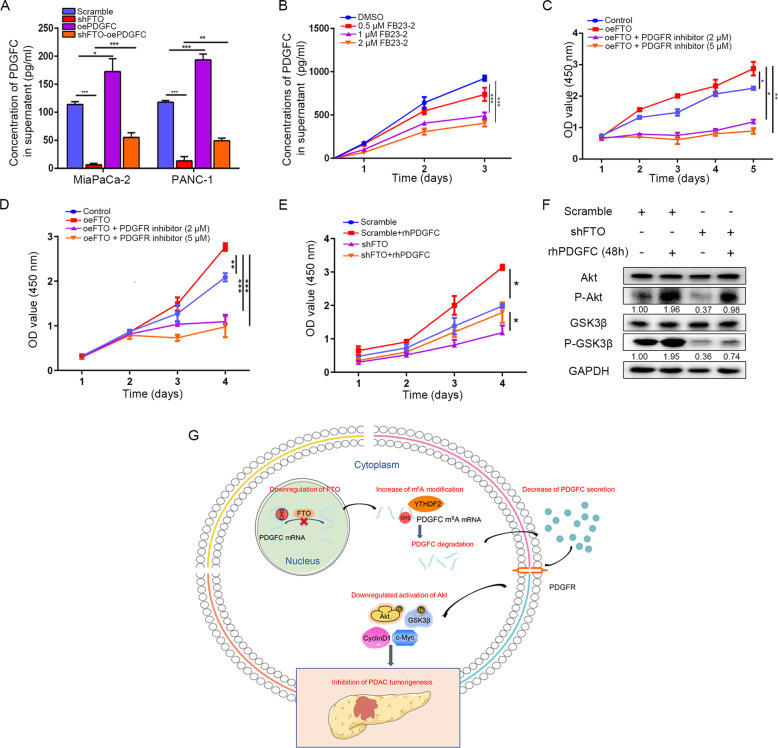
PDGFC is critical to FTO-stimulated Akt signaling. **A** Secreted PDGFC levels were determined in culture supernatants by ELISA. **B** Effect of FB23-2 on PDGFC secretion levels in pancreatic cancer cells. **C** The effect of FTO overexpression alone in CFPAC-1 cells and combination with a PDGFC inhibitor on the number of viable cells was evaluated. **D** The effect of FTO overexpression alone in MiaPaCa-2 cells and combination with a PDGFC inhibitor on the number of viable cells was evaluated. **E** MiaPaCa-2 cells transfected with lentiviruses carrying FTO shRNA or scramble were subjected to CCK8 assay in the presence or absence of rhPDGFC (10 ng/mL). **F** P-Akt, and P-GSK3β expression levels were measured by western blot in the absence or presence of rhPDGFC (10 ng/mL) for 48 h. **G** The graphical explanation of the mechanisms.

We overexpressed PDGFC in FTO stable knockdown cells, and the endogenous level of PDGFC secretion by PDAC cells was detected using ELISA (Fig. 8A). As anticipated, PDGFC released from PDAC cells was decreased when FTO was stably knocked down and rescued when PDGFC was overexpressed. FB23-2, an inhibitor that directly binds to FTO and selectively inhibits FTO m^6^A demethylase activity, indeed inhibited PDGFC secretion in PDAC cells (Fig. 8B). Next, we measured cell viability in the presence of a PDGFR inhibitor. According to the detection of FTO levels in the pancreatic cancer cell lines, we chose CFPAC-1 with the lowest FTO expression level to construct an FTO overexpression model. FTO overexpression in CFPAC-1 cells enhanced proliferation and viability, whereas FTO overexpression in combination with a PDGFR inhibitor demonstrated a stronger inhibitory effect. In addition, we also overexpressed FTO in MiaPaCa-2 cells and repeated these experiments. As shown in Fig. 8C, D, CCK-8 assays demonstrated that FTO overexpression in CFPAC-1 and MiaPaCa-2 cells enhanced proliferation and viability, whereas FTO overexpression in combination with a PDGFR inhibitor demonstrated a stronger inhibitory effect. Besides it, CCK8 analysis of FTO scramble and knockdown cells incubated in DMEM (full medium) treated with or not recombinant human PDGFC (rhPDGFC) (10 ng/ml) was also proved the stimulated proliferative activities of exogenous PDGFC (Fig. 8E). In order to further elucidate signaling pathways induced by PDGFC, we treated MiaPaCa-2 cells with rhPDGFC (10 ng/ml). Notably, 48 h of rhPDGFC pretreatment induced phosphorylation levels of Akt/GSK3β activation in scramble and FTO knockdown conditions (Fig. 8F). Collectively, these results show that autocrine endogenous and exogenous rhPDGFC could promote PDAC cell growth and it was driven part by Akt/GSK3β signaling.

## Discussion

PDAC remains a lethal solid organ malignancy and is expected to become the second leading cause of cancer-related death in the United States of America in the next 20-30 years due to features of the microenvironment that inhibit effective penetration by immunotherapy agents [20]. A growing body of evidence has confirmed the impact of m^6^A on fine-tuning and coordinating gene expression [21]. Alterations in the m^6^A level could influence the activation of proliferative signaling, reprogramming energy metabolism and evading immune destruction. Thus, m^6^A participates in some biological processes in malignant tumor development [22]. In this study, we show that the dysregulation of FTO can affect m^6^A levels in PDAC cells. We then provide evidence that FTO can regulate the growth and proliferation of PDAC through PDGFC via Akt signaling. Moreover, mechanistically, FTO downregulation leads to increased m^6^A modifications in the 3ʹ UTR of PDGFC and then modulates the degradation of its transcriptional level in an m^6^A-YTHDF2-dependent manner (Fig. 8G). These observations reveal interesting new complexities in epitranscriptomic alterations that provide a novel perspective on cancer development and novel interventional therapies.

FTO has been correlated with obesity in humans based on genome-wide association studies since 2007 [23, 24]. Several single-nucleotide polymorphisms (SNPs) in the FTO gene which located in a linkage disequilibrium block is predicted to be link with many cancers. Variation in rs9939609, rs6499640, rs19079260, and rs8050136 in FTO genotype was associated with higher endometrial cancer risk [25, 26]. Furthermore, recent studies have delineated rs9939609 was related to susceptibility to pancreatic cancer but were no significant association with the risk of prostate cancer [27]. Certain FTO SNPs variations are strongly linked to susceptibility to cancers. Moreover, as the first recognized RNA m^6^A demethylase, recent studies have begun to delineate that FTO plays m^6^A-dependent roles in adipogenesis and tumorigenesis [28–30]. Li et al. established the first in vivo animal model to demonstrate the role of FTO in subtypes of acute myeloid leukemia (AML). FTO negatively regulates the expression of Ankyrin Repeat and SOCS Box Containing 2 (ASB2) and Retinoic Acid Receptor Alpha (RARA), a cluster tumor suppressor target gene, by post-transcriptionally modulating its m^6^A abundance and then affecting its stability [31]. However, the roles of FTO in pancreatic cancer progression are not completely understood. Hence, to further illuminate the exact mechanism by which FTO regulates PDAC cell proliferation, we performed high-throughput sequencing. Subsequently, PDGFC was identified as a downstream target of the FTO-mediated m^6^A modification by RNA-seq and m^6^A-seq analyses. Due to the key roles of FTO in cancers, the identification of selective and effective inhibitors targeting FTO hold great therapeutic potential in carcinogenesis. R-2-Hydroxyglutarate (R-2HG), a well-established oncometabolite, could bind directly to the FTO protein and inhibit its m^6^A demethylase level, exhibiting intrinsic antitumor activity in R-2HG-sensitive AML [32]. Subsequently, Huang et al. found that two potential FTO inhibitors, FB23 and FB23-2, could bind to FTO and discriminatingly inhibit the activity of FTO m^6^A demethylase, suppress proliferation and promote the apoptosis of human AML cells [33]. In our study, we used FB23-2 to treat PDAC cells and found that FB23-2 could decrease PDGFC secretion levels. It is possible that the development of specific potent FTO inhibitors could lead to more beneficial outcomes in the treatment of PDAC.

The PDGF family includes four different homodimers (PDGFA, PDGFB, PDGFC, and PDGFD) that play an important role in regulating cell growth, survival and transformation through dimerization by combining with their monomeric forms. Previous studies demonstrated that PDGFC functions as a mitogenic factor with superior activity to PDGFA but similar activity to PDGFB in cells of mesenchymal origin [34, 35]. By binding to platelet-derived growth factor receptors (PDGFRs) and thereby initiating intracellular signaling events, PDGFC regulates a variety of biological processes [36, 37]. Additionally, PDGFC acts directly on tumor cells and promotes their proliferation. Indeed, PDGFC also activates PDGFC-α signal transduction in gastrointestinal stromal tumor cells and manages the expression of slug and downstream targets, stimulating cancer cell proliferation and metastasis in a paracrine manner [38]. Furthermore, when we analyzed public databases and our PDAC patient cohort, we found that PDGFC was significantly correlated with poor OS. It is therefore tempting to hypothesize that PDGFC may also play a role in the development of PDAC.

PDGFC participates in multiple cellular processes by binding specifically to PDGFRs. The stimulated receptors function by interacting with the complex Ras/MAPK signaling cascade and activating various downstream targets, including the Akt pathways [39]. m^6^A-seq screening performed in scramble and FTO-knockdown cells revealed that Akt signaling was activated to a large extent following FTO silencing. Increasing evidence suggests that the Akt signaling pathway plays a key role in various cellular events, such as cell cycle progression, proliferation and transcriptional regulation. In malignant breast tumors, PDGFC mediates antiapoptotic effects through Akt/Bad phosphorylation, underlining the important role of PDGFC in cell growth and survival through resident macrophages in tumors [39]. Based on this evidence, we propose that this molecule might be a downstream effector of PDGFC signaling. As expected, FTO knockdown decreased the activation of phosphorylated Akt and its downstream modulators, whereas PDGFC overexpression rescued this tendency. Subsequently, an Akt inhibitor (MK2206) abolished the expression of these genes in PDGFC-overexpressing cells. PDGFS and PDGFR overexpression serve as treatment markers in numerous cancers. Oncological modifications of the PDGFR/PDGF system affect the extracellular, transmembrane and tyrosine kinase domains [40]. In PDAC, this system has a wide array of treatment possibilities in stroma-targeting therapy. The ability to target chemotherapeutic agents to the stroma is due to the biological effect of PDGFR/PDGF on vessel normalization that invariably enhances drug efficacy, thereby counteracting anticancer drug resistance [41]. Imatinib, an oral multitarget inhibitor of tyrosine kinases, including PDGFR, decreases the interstitial fluid pressure in tumors and enhances the uptake rate of antibodies in chemotherapeutics [42]. Similarly, sorafenib, another small-molecule inhibitor that inhibits a number of tyrosine kinases, including PDGFR, is active and significantly suppresses tumor cell proliferation and angiogenesis [43]. The diverse therapeutic targets of the PDGFR/PDGF system could be harnessed to develop treatment regimens that are safer and more efficacious in PDAC.

## Conclusions

In summary, our study revealed reduced levels of m^6^A methylation in pancreatic cancer caused by the dysregulation of FTO, the key m^6^A demethylase modulator. The combination of FTO and PDGFC could serve as a prognostic tool and treatment response marker. The potential of therapeutic targets of FTO/PDGFC/PDGFR could be used to develop treatment regimens that are efficacious in the treatment of PDAC. These findings provide novel insight into the molecular mechanisms of PDAC tumorigenesis regulated by m^6^A modification.

## Materials and methods

### Cell culture and reagents

The human PDAC cell lines Capan-1, MiaPaCa-2, BxPC-3, SW1990, CFPAC-1, ASPC-1, PANC-1, 239 T and HPDE were commercially obtained from the American Type Culture Collection and certified by DNA fingerprinting. ASPC-1 and BxPC-3 cells were cultured in RPMI 1640. CFPAC-1 and Capan-1 cells were cultured in IMDM, and other pancreatic ductal cancer cell lines were cultured in DMEM. Cell media was supplemented with 10% fetal bovine serum and 100 U/mL penicillin, and cells were grown in a humidified incubator at 37 °C with 5% CO_2_ and assessed for mycoplasma contamination by PCR every three months. HPDE cells were grown in complete keratinocyte serum-free medium containing epidermal growth factor (5 ng/mL) and bovine pituitary extract (50 µg/mL). Actinomycin D (Dactinomycin), Akt inhibitor (MK2206), FTO inhibitor (FB23-2) and PDGFR inhibitor (PDGFR inhibitor 1) were obtained from Selleck (Houston, TX, USA).

### Bioinformatics analysis

The gene expression profile datasets GSE15471, GSE62452, GSE71989, GSE16515 and GSE32676 were downloaded from the GEO database. To validate the expression level of the m^6^A regulators and PDGFC in cancerous tissues compared with paracarcinoma tissues, we used the GEO database for external validation. First, the GSE15471 database was used to confirm the expression. Then, we further validated these results using related array data from other GEO datasets. GSE62452 and TCGA were used to identify the expression differences in m^6^A regulators. GSE71989 was used to detect YTHDF2 expression differences. GSE71989, GSE16515 and GSE32676 were applied to validate the expression differences of PDGFC. Data from the GEO database or RNA sequencing (RNA-seq) were investigated with the edge R package of R (V3.3, http://www.bioconductor.org). PDAC transcriptome profiles with clinical data were obtained from The Cancer Genome Atlas (TCGA, https://portal.gdc.cancer.gov), GTEX data (http://www.gtexportal.org), Array Express (https://ebi.ac.uk/arrayexpress/) and the International Cancer Genome Consortium (ICGC; https://dcc.icgc.org/).

### Clinical information on patients with PDAC

Clinical information was obtained from TMAs that were acquired from patients who underwent surgical resection and were clinically diagnosed with PDAC at FUSCC between 2010 and 2012. Two experienced pathologists diagnosed the samples and performed postoperative follow-ups. Samples from another 84 PDAC patients who underwent surgical resection were used for RNA extraction and quantitative real-time PCR (qRT-PCR) analysis to construct the FUSCC validation set. None of the patients included in our study received any anticancer treatment, including chemotherapy and radiotherapy, before surgical resection.All patients received strict postoperative follow-up care. All experiments were accomplished with approval from the Clinical Research Ethics Committee of FUSCC (Project identification code: 050432-4-1805 C).

### qRT-PCR

Total RNA was isolated from clinical tumor samples and human PDAC cells with TRIzol reagent (Invitrogen, Carlsbad, CA, USA) and subsequently reverse transcribed into cDNA with a Prime Script RT Reagent Kit (TaKaRa, Shanghai, China) according to the manufacturer’s instructions. The 2^−ΔΔCt^ method was applied to calculate mRNA expression levels, and the levels were normalized to that of GAPDH. The specific primer sequences used in this study are shown in Supplementary Table S1.

### Western blot

Western blotting was performed as described in our previous study [44]. The antibodies used in the present study included those against FTO (1:1000; Abcam), GAPDH (1:20000; Proteintech), Akt (1:2000; Abcam), phosphorylated-Akt^Ser473^ (1:2000; Abcam), phosphorylated-GSK3β (1:1000; Cell Signaling Technology), Cyclin D1 (1:20000; Abcam), c-Myc (1:1000; Abcam), and PDGFC (1:2000; Abnova). The grayscale of indicated protein was quantified by image analysis software (ImageJ 1.51e, NIH Image).

### Enzyme-linked immunosorbent assay

Enzyme-linked immunosorbent assay (ELISA) was performed using the Human PDGFC simple step ELISA Kit (Abcam) depending on the manufacturer’s description. Cell culture supernatants were extracted and filtered through a 0.45-μm Filter Unit (Steriflip, Millipore). 450-nm absorbance for sample was calculated by an ELISA reader and interpolated with a standard curve.

### Immunohistochemistry staining

IHC staining with antibodies against FTO (1:200; Abcam), Ki-67 (1:400; Cell Signaling Technology) and PDGFC (1:200; Abnova) was performed to determine protein expression based on previously described methods. The FTO and Ki-67 staining levels were evaluated by multiplying the positivity (no positive cells: 0; positive cells <10%: 1; 10% ≤ positive cells <50%: 2; 50%≤ positive cells <80%: 3; or ≥80%: 4) by the intensity scores (0, negative; 1, weakly positive; 2, moderately positive; and 3, strongly positive). Then, the patients were subdivided into two groups (scores <6, low expression; and ≥6, high expression).

### RNA m^6^A quantification

Total RNA from clinical tumor samples and human PDAC cells was isolated with TRIzol reagent (Thermo Fisher, USA) according to the manufacturer’s instructions. RNA quality was analyzed on a NanoDrop 3000. An m^6^A RNA Methylation Assay Kit (Abcam, MA, USA) was used to detect the m^6^A level in total RNA. The m^6^A levels were quantified colorimetrically by acquiring the absorbance at a wavelength of 450 nm, and calculations were performed according to the standardized curve.

### In vitro cell proliferation assays

For the CCK-8 assay (Gaithersburg, MD, USA), cells were seeded at a density of 2,000 cells per well in 96-well plates, and cell viability was assayed. The microplates were incubated at 37 °C for 2 h, and the absorbance was acquired at 450 nm. For the clonogenic assay, 1,000 cells were seeded and then incubated in 6-cm^2^ cell dishes for two weeks. The cells were fixed with 4% paraformaldehyde and stained with 0.1% crystal violet (Sigma, St. Louis, MO, USA). Colonies were counted using a light microscope until the colonies became visible. For the EdU assay, dissociated cells in logarithmic growth phase were exposed to the corresponding concentration of EdU reagent for 2 h at 37 °C. Then, the cells were washed with phosphate-buffered saline (PBS) and fixed with 4% paraformaldehyde for 20 min. Ultimately, the DNA contents of the cells were stained with 4′,6-diamidino-2-phenylindole (DAPI) for 30 min, and images were obtained under a fluorescence microscope (OLYMPUS, Tokyo, Japan).

### Flow cytometry analysis

The cells were stained with propidium iodide (PI) to conduct cell cycle analysis using a cell cycle staining kit (Beyotime Biotechnology, China) according to the manufacturer’s protocol and counted using a FACSCalibur flow cytometer.

### Confocal microscopy

Cells transfected with PDGFC-overexpressing lentivirus were cultured in glass bottom cell culture dishes (Nest) with cover slips at a density of 1 × 10^3^ cells per well. The cells were first incubated with antibodies specific for PDGFC (1:100; Abcam) and then with goat anti-rabbit IgG (Alexa Fluor 594, Invitrogen). The changes were assessed using confocal microscopy (LEICA SP5, Leica Biosystems, USA).

### m^6^A sequencing (m^6^A-seq)

Total RNA from transfected pancreatic cancer cells was extracted using TRIzol reagent (Invitrogen). Then, mRNA-seq and m^6^A-seq were performed simultaneously (LC Biotechnology Co., Ltd., Hangzhou, China). For mRNA-seq, 1 μg of total RNA representing a specific type of adipose tissue was subjected to poly(A) mRNA isolation with Dynabeads Oligo-dT (Thermo Fisher, cat.25-61005). A cDNA library was constructed based on the protocol for the TruSeq RNA Library Prep Kit v2 (Illumina). Ultimately, we performed 2 × 150 bp paired-end (PE150) sequencing on an Illumina NovaSeq 6000. For m^6^A-seq, the cleaved RNA fragments were incubated with the m^6^A antibody (Synaptic Systems, cat. 202003) in immunoprecipitation buffer (750 mM NaCl, 50 mM Tris-HCl and 0.5% Igepal CA-630), and PE150 sequencing was performed on an Illumina NovaSeq™ 6000.

### RNA immunoprecipitation (RIP) and qRT-PCR assays

RIP assays were performed using an EZ-Magna RIPTM RNA-Binding Protein Immunoprecipitation Kit (Millipore Sigma, cat. 17-700) according to the manufacturer’s instructions. Briefly, cell supernatants were lysed with radioimmunoprecipitation assay lysis buffer containing an RNase inhibitor and proteases and then incubated with YTHDF2 antibody beads overnight at 4 °C. Proteinase K (Millipore Sigma, cat. 71049) was added, and the mixture was incubated at 65 °C for 30 min with occasional shaking of the beads. Finally, total RNA was isolated using TRIzol reagent.

### Luciferase reporter and mutagenesis assays

The dual-luciferase vector pmirGLO was purchased from Promega (Promega, cat. C838A). The 3ʹ UTR of PDGFC was amplified by PCR using genomic DNA from 293 T cells as a template. A clone with a sequence that was identical to the NCBI reference sequence NM_016205 was cloned into the pmirGLO vector at the SacI and SalI restriction sites. Two putative m^6^A recognition sites were identified in the 3ʹ UTR. Mutagenesis from A to T was generated using the QuikChange II Site-Directed Mutagenesis Kit (Agilent, USA) according to the manufacturer’s instructions. Luciferase activity was measured with a Dual-Luciferase Reporter Gene Assay Kit (Promega, cat. E1910). The activity of firefly luciferase was normalized to that of Renilla luciferase to evaluate reporter expression efficiency.

### Animal models

BALB/c female nude mice (4–6 weeks of age, 18–20 g, Shanghai SLAC Laboratory Animal Co., Ltd.) were housed in sterile, filter-capped cages. These mice were randomly divided into two subgroups (*n* = 5/group). The right flanks of mice were injected subcutaneously with 2 × 10^6^ MiaPaCa-2 cells stably expressing shFTO and a scrambled shRNA in 100 μL PBS. Tumors were measured using an external caliper once per week, and tumor volume was calculated with the formula: (length × width^2^)/2. The mice were euthanized at the 5th week, and the tumors were surgically dissected for IHC examination and qRT-PCR analysis. Tumor weights were measured at the 5th week after subcutaneous transplantation. The animal experiment in this study was performed strictly in accordance with the guidelines of the Committee on the Ethics of Animal Experiments of Fudan University.

### Statistical analysis

Statistical analysis was performed in R (version 3.6.2, www.r-project.org), SPSS version 22 (SPSS Inc., Chicago, IL, USA) and GraphPad Prism v. 7.01 (GraphPad Software, La Jolla, CA, USA). Categorical variables were analyzed using the χ^2^ test or Fisher’s exact test. Continuous variables were analyzed using Student’s *t*-test for paired samples. Spearman correlation analysis was used to determine the association between the expression of genes in the signature and that of FTO and PDGFC. Kaplan–Meier curves and log-rank tests were used to determine the overall survival (OS) rates and curves of PDAC patients. Results are means ± SEM from three independent experiments performed in duplicate. All statistical tests were two-sided, and significant differences were considered significant at *P* < 0.05, *P* < 0.01 and *P* < 0.001.

## Supplementary information

supplementary figure legend

Table.S1

Fig.S1

Fig.S2

Fig.S3

Fig.S4

Fig.S5

Fig.S6
