# Supplementary material for: RNA N6-methyladenosine demethylase FTO promotes pancreatic cancer progression by inducing the autocrine activity of PDGFC in an m6A-YTHDF2-dependent manner
Source: Oncogene. 2022 Apr 14;41(20):2860–72. doi: 10.1038/s41388-022-02306-w (PMC9106577; doi:10.1038/s41388-022-02306-w)
Supplement: Supplementary file 1 — supplementary figure legend [file 41388_2022_2306_MOESM1_ESM.docx]

**Fig. S1 FTO overexpression significantly increased the proliferation rate. A.** FTO overexpression efficiency was confirmed at the protein level. **B.** Increased FTO expression augmented the colony formation capacity of CFPAC-1 cells. **C.** FTO overexpression enhanced proliferation, as reflected by the CCK-8 proliferation assay. **D.** EdU incorporation assays observed the effects of FTO overexpression on cell proliferation.

**Fig. S2** **Immunohistochemical staining of subcutaneous and the efficiency of siRNA knockdown. A-B.** Akt/GSK3β phosphorylation levels were determined in xenograft tumor tissue sections using IHC. **C.** PDGFC expression levels were determined in xenograft tumor tissue sections using IHC. **D.** 293T cells were pretreated with small interfering RNA (siRNA) targeting FTO for 48-72 h. Then, FTO mRNA and protein levels were detected.

**Fig. S3** **Differentially expressed genes from MeRIP-seq and literature were validated by qRT–PCR.**

**Fig. S4 YTHDF2 is overexpressed in PDAC patients. A.** YTHDF2 mRNA expression levels in TCGA pancreatic cancer tumor tissue and matching normal tissue from TCGA and GTEx datasets. Data were obtained from GEPIA (http://gepia.cancer-pku.cn/). **B-C.** YTHDF2 mRNA levels in the GSE15471 and GSE71989 PDAC cohorts. **D.** Immunohistochemistry staining showed the upregulation level of YTHDF2 in tumor tissues.

**Fig. S5 PDGFC is overexpressed in PDAC patients. A.** PDGFC mRNA expression levels in TCGA pancreatic cancer tumor tissue and matching normal tissue from TCGA and GTEx datasets. Data were obtained from GEPIA (http://gepia.cancer-pku.cn/). **B-E.** PDGFC mRNA levels in the GSE16515, GSE32676, GSE15471 and GSE71989 PDAC cohorts. **F–I.** PDGFC expression levels in the context of different mutation statuses of CDKN2A, KRAS, SMAD4 and TP53 in TCGA dataset.

**Fig. S6 PDGFC overexpression significantly increased PDAC cell proliferation. A.** PDGFC overexpression efficiency was confirmed at the protein level. **B.** Expression and localization of PDGFC using immunofluorescence staining. **C.** Increased PDGFC expression facilitated the colony formation capacity of PANC-1 and MiaPaCa-2 cells. **D.** PDGFC overexpression promoted proliferation, as reflected by the CCK-8 proliferation assay. **E.** EdU incorporation assays showed the effects of PDGFC overexpression on cell proliferation.
