# Supplementary material for: RNA N6-methyladenosine demethylase FTO promotes pancreatic cancer progression by inducing the autocrine activity of PDGFC in an m6A-YTHDF2-dependent manner
Source: Oncogene. 2022 Apr 14;41(20):2860–72. doi: 10.1038/s41388-022-02306-w (PMC9106577; doi:10.1038/s41388-022-02306-w)
Supplement: Supplementary file 2 — Table.S1 [file 41388_2022_2306_MOESM2_ESM.docx]

Table.S1. The sequences of the primers.

| Primer | Sequence |
| --- | --- |
| FTO-Forward | GCTGCTTATTTCGGGACCTG |
| FTO-Reverse | AGCCTGGATTACCAATGAGGA |
| PDGFC-Forward | CAGGGTTCTGCATCCACTACA |
| PDGFC-Reverse | CTTCTAAGTCCAACTGCCATCT |
| YTHDF2-Forward | CCTTAGGTGGAGCCATGATTG |
| YTHDF2-Reverse | TCTGTGCTACCCAACTTCAGT |
| GAPDH-Forward | GGAGCGAGATCCCTCCAAAAT |
| GAPDH-Reverse | GGCTGTTGTCATACTTCTCATGG |
| PTEN-Forward | TTTGAAGACCATAACCCACCAC |
| PTEN-Reverse | ATTACACCAGTTCGTCCCTTTC |
| PHLPP2-Forward | ATGGAGCAGACACTACCACTG |
| PHLPP2-Reverse | GCAAAGGACGAGATGTAAGTCA |
| mTOR-Forward | TCCGAGAGATGAGTCAAGAGG |
| mTOR-Reverse | CACCTTCCACTCCTATGAGGC |
| PRR5-Forward | TCAGTGTGAAGCTAGAGGATGC |
| PRR5-Reverse | GTATGGCGACACCACCTTCT |
| PRR5L-Forward | CGGCTGTTGAAGAGTGAACTT |
| PRR5L-Reverse | GCAGGGTAGGGAGAGTCTCAG |
| ITGB5-Forward | GGAAGTTCGGAAACAGAGGGT |
| ITGB5-Reverse | CTTTCGCCAGCCAATCTTCTC |
| CREB3L1-Forward | GCACCTGGACCACTTTACGG |
| CREB3L1-Reverse | AGCACAGGGTCATCAAAGAAG |
| COL6A2-Forward | GACTCCACCGAGATCGACCA |
| COL6A2-Reverse | CTTGTAGCACTCTCCGTAGGC |
| EFNA5-Forward | CGCTACGCTGTCTACTGGAAC |
| EFNA5-Reverse | TTCTGGGACGGAGTCCTCATA |
