# Supplementary figures and images for: RNA N6-methyladenosine demethylase FTO promotes pancreatic cancer progression by inducing the autocrine activity of PDGFC in an m6A-YTHDF2-dependent manner
Source: Oncogene. 2022 Apr 14;41(20):2860–72. doi: 10.1038/s41388-022-02306-w (PMC9106577; doi:10.1038/s41388-022-02306-w)

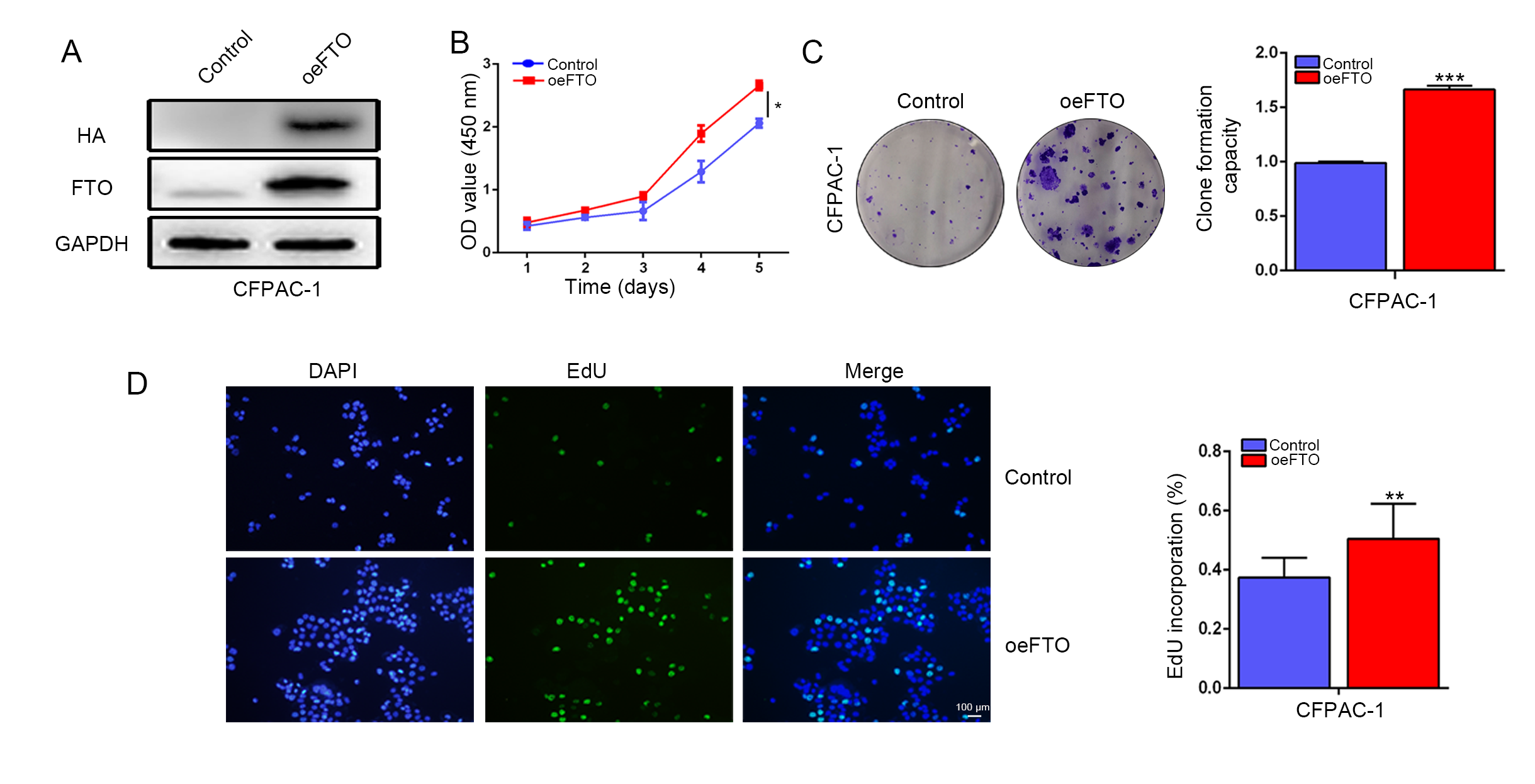

Supplement: Supplementary file 3 — Fig.S1 [file 41388_2022_2306_MOESM3_ESM.tif]

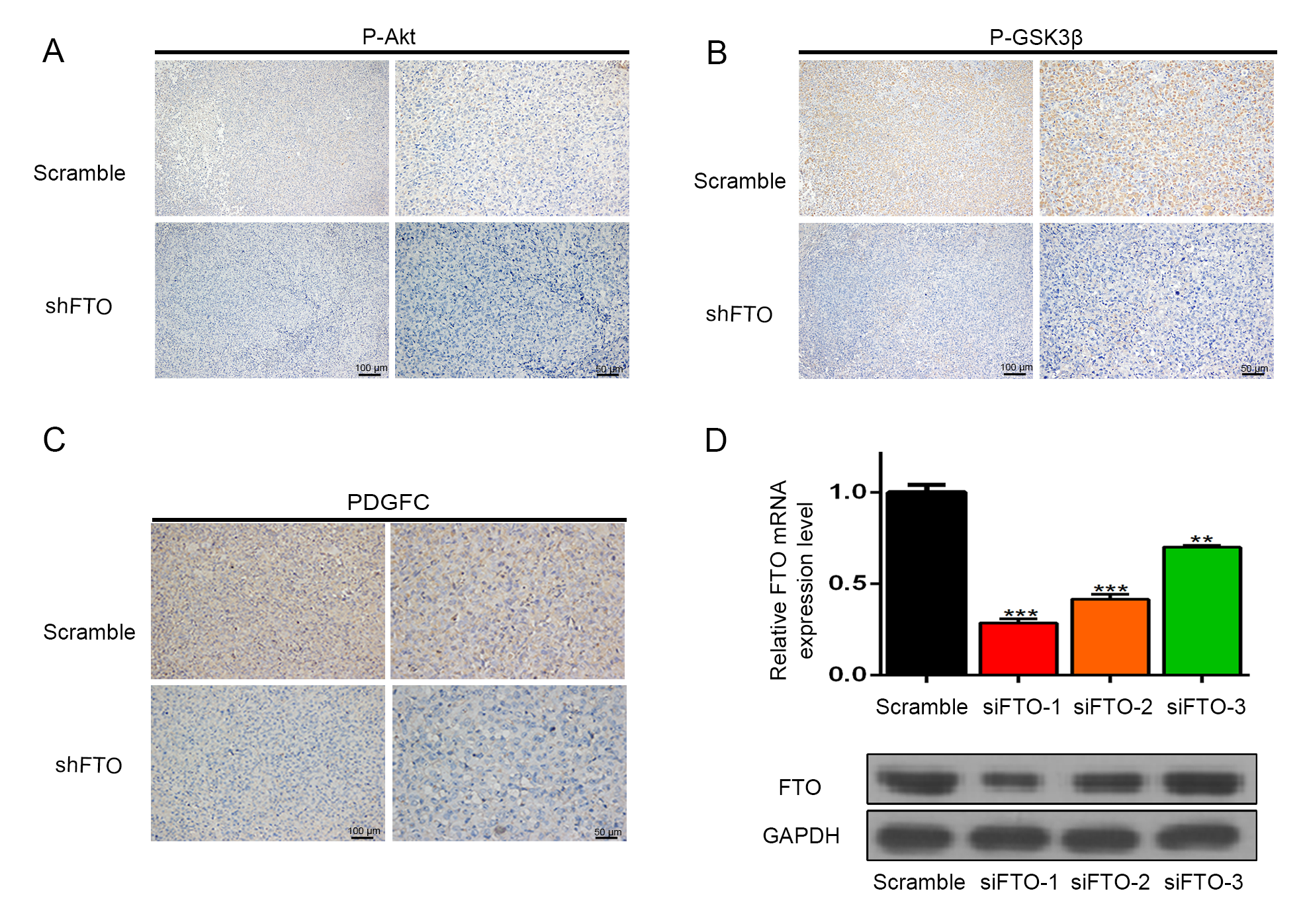

Supplement: Supplementary file 4 — Fig.S2 [file 41388_2022_2306_MOESM4_ESM.tif]

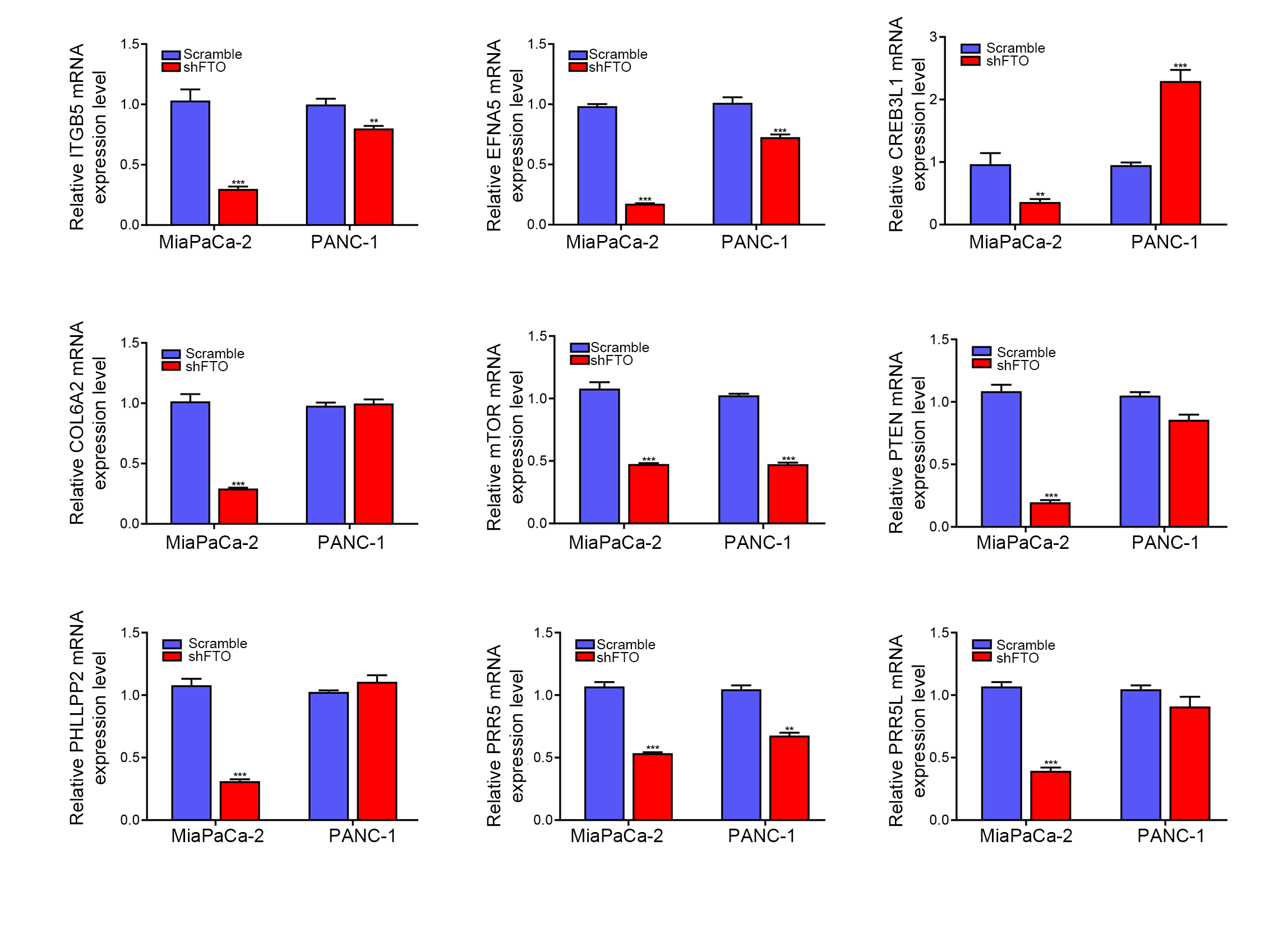

Supplement: Supplementary file 5 — Fig.S3 [file 41388_2022_2306_MOESM5_ESM.tif]

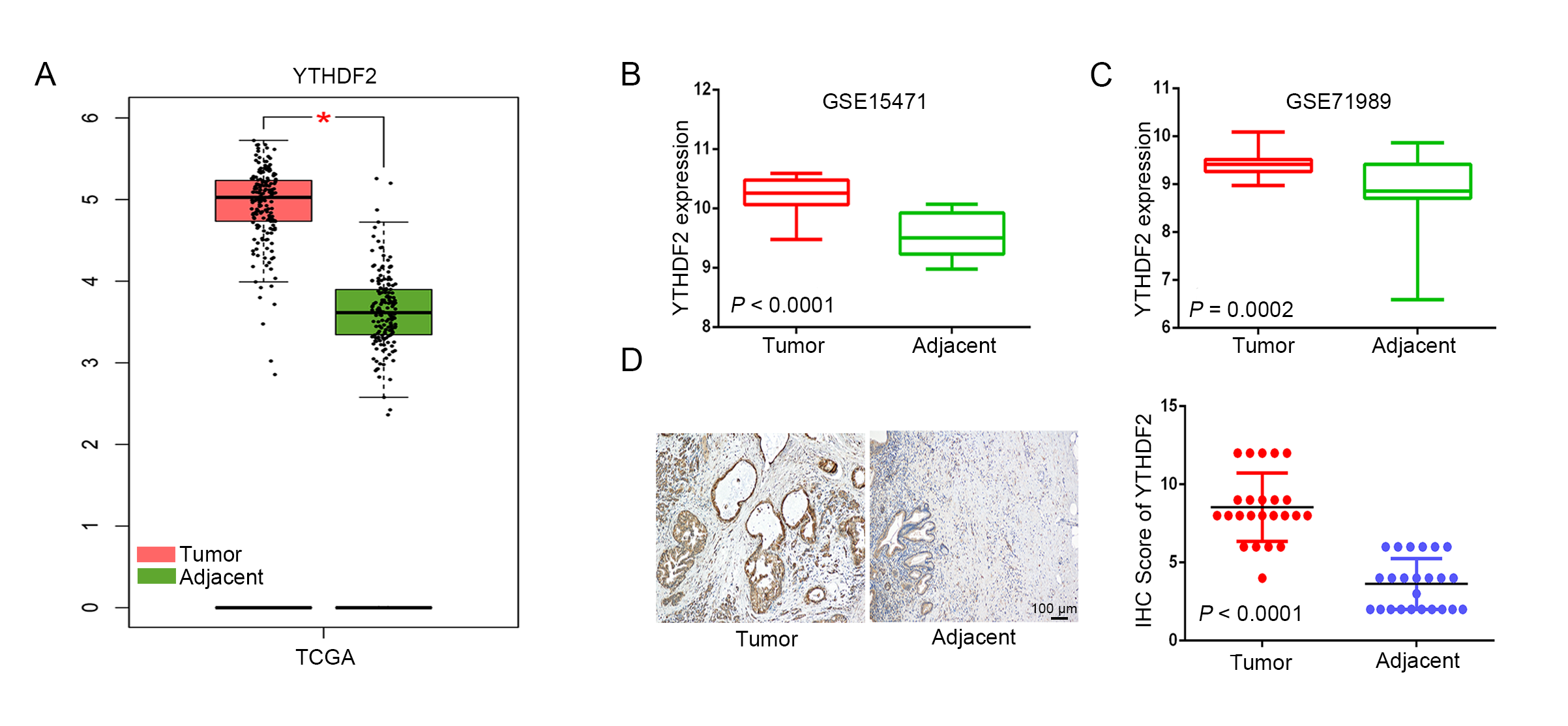

Supplement: Supplementary file 6 — Fig.S4 [file 41388_2022_2306_MOESM6_ESM.tif]

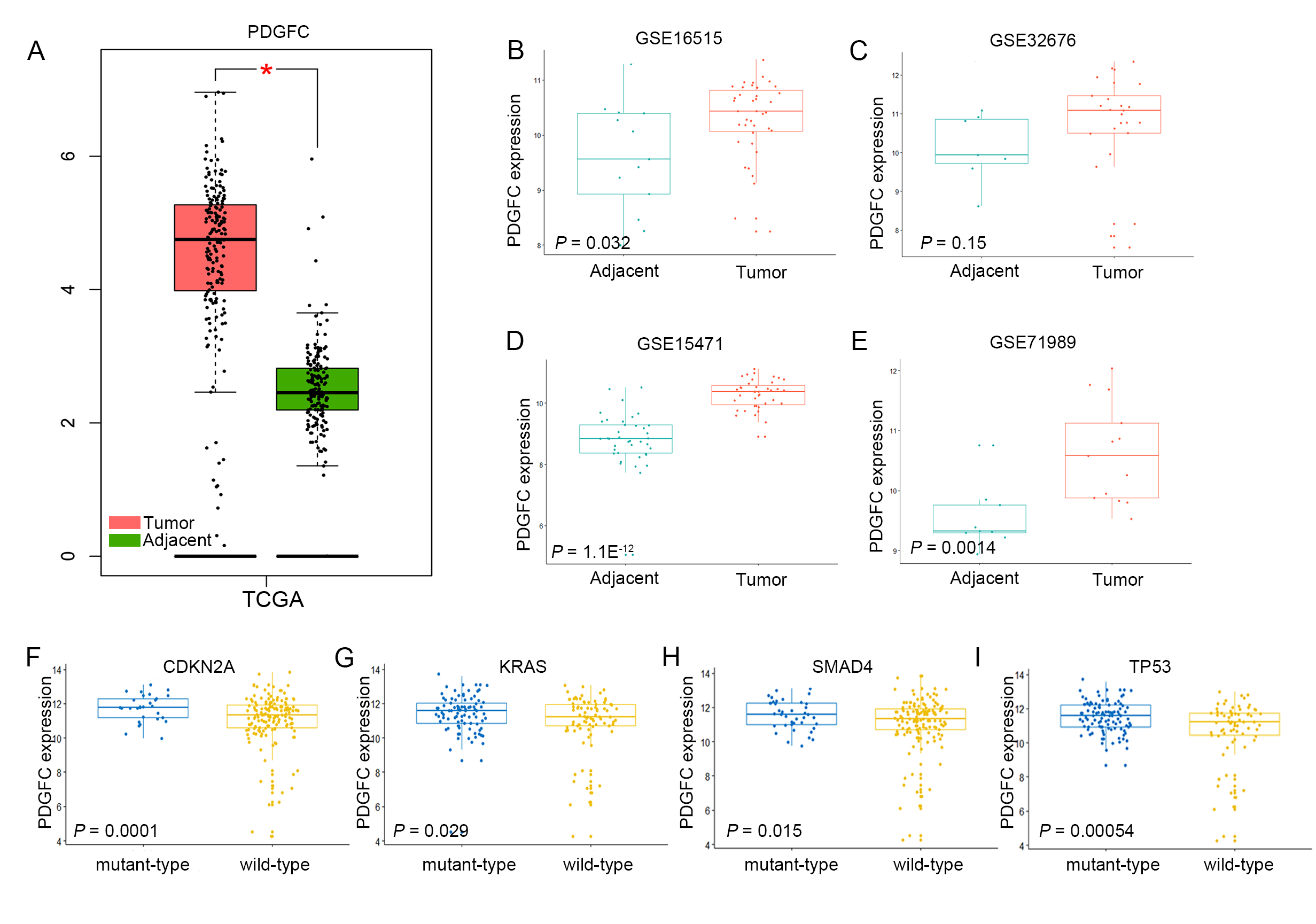

Supplement: Supplementary file 7 — Fig.S5 [file 41388_2022_2306_MOESM7_ESM.tif]

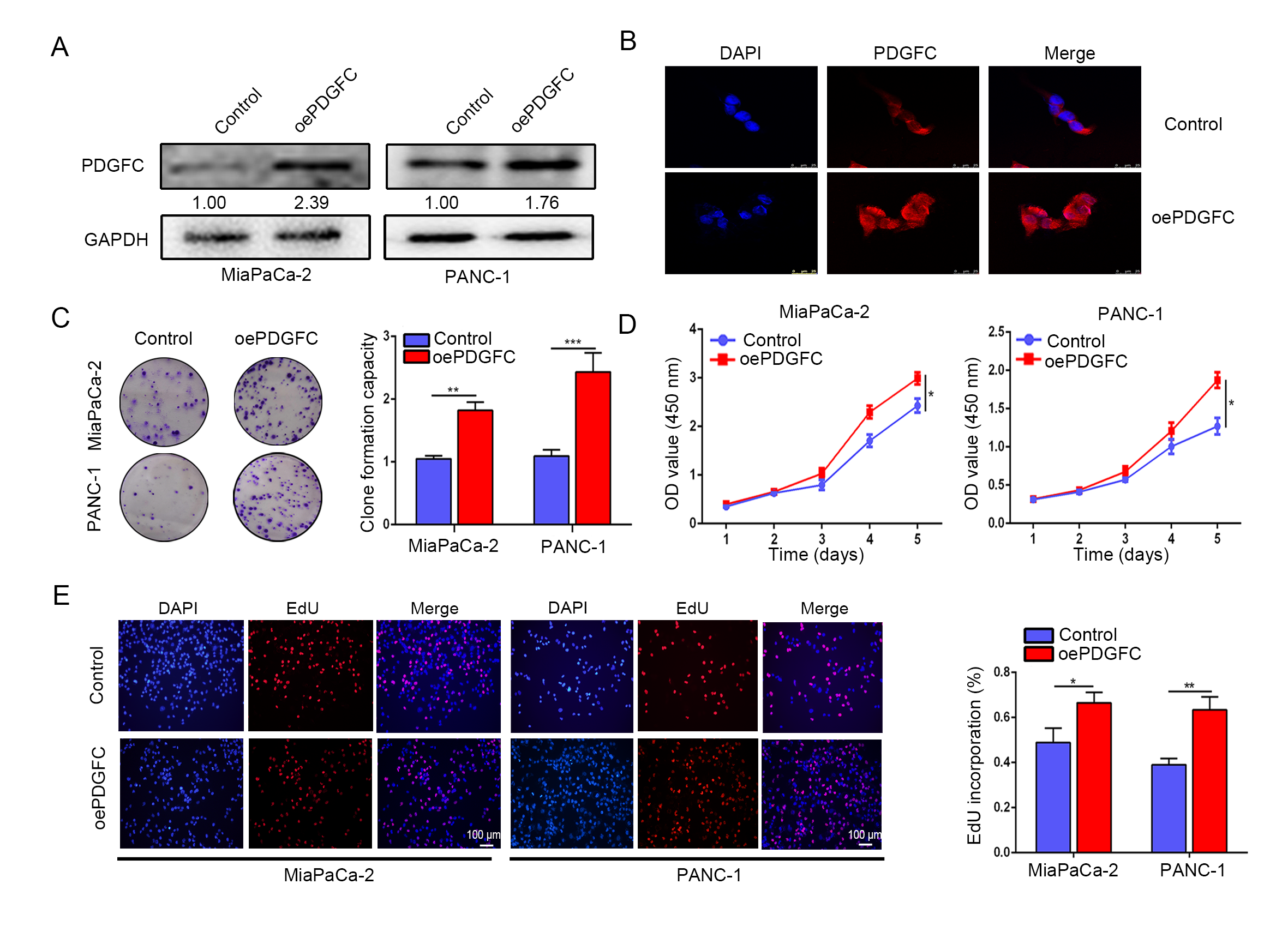

Supplement: Supplementary file 8 — Fig.S6 [file 41388_2022_2306_MOESM8_ESM.tif]
